# Effects of Resveratrol on Nonmelanoma Skin Cancer (NMSC): A Comprehensive Review

**DOI:** 10.1002/fsn3.4555

**Published:** 2024-10-24

**Authors:** Mohammad Yasin Zamanian, Taha Shahbazi, Syeda Wajida Kazmi, Beneen M. Hussien, Abhishek Sharma, Maytham T. Qasim, Ahmed Hjazi, Ibrohim B. Sapaev, Ayda Nouri Danesh, Niloofar Taheri, Maryam Golmohammadi

**Affiliations:** ^1^ Department of Pharmacology and Toxicology, School of Pharmacy Hamadan University of Medical Sciences Hamadan Iran; ^2^ Department of Dermatology, School of Medicine, Sina Research Center Hamadan University of Medical Sciences Hamadan Iran; ^3^ Depatment of Dermatology, Razi Hospital Tehran University of Medical Sciences Tehran Iran; ^4^ Chandigarh Pharmacy College, Chandigarh Group of Colleges Mohali Punjab India; ^5^ Medical Laboratory Technique College The Islamic University Najaf Iraq; ^6^ Medical Laboratory Technique College The Islamic University of Al Diwaniyah Al Diwaniyah Iraq; ^7^ Medical Laboratory Technique College The Islamic University of Babylon Babylon Iraq; ^8^ Department of Medicine National Institute of Medical Sciences, NIMS University Rajasthan Jaipur India; ^9^ Department of Anesthesia, College of Health and Medical Technololgy Al‐Ayen University Thi‐Qar Nasiriyah Iraq; ^10^ Department of Medical Laboratory Sciences, College of Applied Medical Sciences Prince Sattam Bin Abdulaziz University Al‐Kharj Saudi Arabia; ^11^ Tashkent Institute of Irrigation and Agricultural Mechanization Engineers, National Research University Tashkent Uzbekistan; ^12^ New Uzbekistan University Tashkent Uzbekistan; ^13^ School of Medicine Shahroud University of Medical Sciences Shahroud Iran; ^14^ School of Medicine Shahid Beheshti University of Medical Sciences Tehran Iran

**Keywords:** A431 cell line, apoptosis, nonmelanoma skin cancer, oxidative stress, resveratrol

## Abstract

Nonmelanoma skin cancer (NMSC) represents the most prevalent form of skin cancer globally, with basal cell carcinoma (BCC) and squamous cell carcinoma (SCC) being the most common types. The search for effective chemopreventive and therapeutic agents has led to the exploration of natural compounds, among which resveratrol (RES), a polyphenolic phytoalexin found in grapes, berries, peanuts, and red wine, has garnered significant attention. This comprehensive review aims to elucidate the effects of RES on NMSC, focusing on its mechanisms of action, efficacy in preclinical studies, and potential as a chemopreventive and therapeutic agent. RES exhibits promising chemopreventive and antineoplastic capabilities against NMSC through various mechanisms, including the induction of apoptosis, inhibition of cell proliferation, modulation of oxidative stress, and anti‐inflammatory activities. Studies have demonstrated that RES can significantly enhance the effectiveness of traditional chemotherapeutic agents, such as 5‐fluorouracil (5‐FU), by inhibiting cellular proliferation and inducing apoptosis in cancerous cells. Furthermore, resveratrol's antioxidant properties may mitigate the impact of reactive oxygen species (ROS) triggered by UV exposure, thus reducing DNA damage and mutations associated with skin cancer development. In vitro and in vivo experiments have shown that RES can effectively hinder the growth and spread of various tumor cell types, including human cutaneous SCC A431 cells, and induce apoptosis. The development of advanced delivery systems, such as nanostructured lipid carriers and liposomes, has been recognized for their potential to enhance the therapeutic effects of RES, particularly its anticancer properties. In conclusion, RES presents a viable candidate for the prevention and treatment of NMSC, owing to its multifaceted mechanisms of action, including its ability to regulate oxidative stress, trigger apoptosis, and inhibit proliferation. However, further clinical studies are required to fully understand its effectiveness and safety in humans, as well as to optimize delivery methods for improved bioavailability and therapeutic outcomes.

## Graphical abstract

Resveratrol (RES), a natural phytoalexin found in grapes and other plants, has shown promising chemopreventive and antineoplastic properties against nonmelanoma skin cancer (NMSC). It operates through various mechanisms, including the modulation of oxidative stress, induction of apoptosis, and inhibition of cell proliferation. This review highlights the potential of RES, particularly when combined with 5‐fluorouracil (5‐FU) and delivered through advanced lipid–nanocarrier systems, to enhance the effectiveness of NMSC treatments.

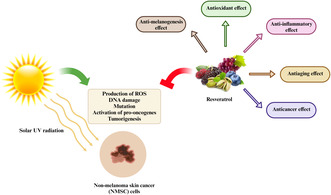

**Abbreviations**
ABCB1: adenosine 5′‐triphosphate–binding cassette subfamily B member 1
ALA: 5‐aminolevulinic acid
CAT: catalase
CREB: cAMP response element‐binding protein
FDA: Food and Drug Administration
GSH: glutathione
HIV: human immunodeficiency virus
IAPs: inhibitor of apoptosis proteins
IL‐10: interleukin 10
IL‐6: interleukin 6
MAPK/ERK: mitogen‐activated protein kinase/extracellular‐signal‐regulated kinase
mTORC2: mammalian target of rapamycin complex 2
PDT: photodynamic therapy
PTEN: phosphatase and tensin homolog
Smac/DIABLO: second mitochondria‐derived activator of caspase/direct inhibitor of apoptosis‐binding protein with low pI
SOD: superoxide dismutase
TGF‐β2: transforming growth factor‐beta 2
TNF‐α: tumor necrosis factor α
TPA: tetradecanoylphorbol acetate
VEGF: vascular endothelial growth factor

## 1 Introduction

Skin cancer (SC) is a prevalent form of human neoplasm, leading to rising morbidity and mortality rates and imposing significant socioeconomic burdens on patients and their families (Al‐Tamimi et al. 2022). Nonmelanoma skin carcinomas (NMSCs), including basal cell carcinoma (BCC) and squamous cell carcinoma (SCC), are the most common forms of global malignancies. However, melanoma is acknowledged as the most aggressive type of SC (Khan et al. 2022).

However, the precise incidence of NMSC remains uncertain. According to the World Health Organization (WHO), approximately 2–3 million cases of malignant skin tumors are diagnosed each year, although it is likely that this number is an underestimate. A recent study provides additional evidence of this underreporting, with an estimated 3.3 million individuals affected by 5.4 million cases of NMSC, including instances where individuals may have multiple skin tumors (Faur et al. 2023).

Furthermore, the GLOBOCAN project, conducted by the International Agency for Research on Cancer, reported a global incidence of 1,198,073 new cases of NMSC (excluding basal cell carcinoma) in 2020. This number was approximately four times higher than the number of new cases of malignant melanoma, which amounted to 324,635 (Sung et al. 2021). The risk of developing NMSC is influenced by various factors, including age, gender, and type of immunosuppressive therapy in transplant patients (Bašić‐Jukić et al. 2022). Men have been found to have a 50% higher risk of developing NMSC compared to women (Bašić‐Jukić et al. 2022). While NMSC generally has a low mortality rate, advanced cases can significantly impair daily functioning, especially in elderly patients (Chicheł et al. 2023). About 5% of skin cancer patients are diagnosed with an advanced stage, which can lead to more serious complications and potentially higher mortality rates (Chicheł et al. 2023).

The cellular and molecular pathogenesis of NMSC primarily involves cumulative ultraviolet (UV) radiation exposure, which leads to DNA damage and mutations in skin cells (Didona et al. 2018). BCC often involves mutations in the Hedgehog signaling pathway, while squamous cell carcinoma SCC is frequently associated with mutations in the p53 tumor suppressor gene (Jee et al. 2015; Bakshi et al. 2017). A study has revealed that aging significantly affects the response of DNA damage in UV‐irradiated skin, with a specific emphasis on the role of insulin‐like growth factor‐1 (IGF‐1) (Kemp, Spandau, and Travers 2017). The reduced production of IGF‐1 by senescent fibroblasts in the dermis of geriatric skin creates an environment that negatively affects how epidermal keratinocytes respond to UVR‐induced DNA damage (Kemp, Spandau, and Travers 2017). Specifically, two principal components of the cellular response to DNA damage—nucleotide excision repair and DNA damage checkpoint signaling—are partially defective in keratinocytes with inactive IGF‐1 receptors (Kemp, Spandau, and Travers 2017). Interestingly, recent research has also highlighted the potential involvement of vitamin D in NMSC pathogenesis, with high vitamin D levels associated with increased NMSC incidence, possibly due to its direct correlation with increased sun exposure (Seretis, Bounas, and Sioka 2023). The role of human papillomaviruses (HPV) in NMSC development has gained attention, particularly in patients with Epidermodysplasia verruciformis (EV), where specific EV/cutaneous HPV types belonging to beta‐ and gamma‐papillomaviruses have been implicated (Nindl, Gottschling, and Stockfleth 2007). These HPV types may act as cofactors in association with UV radiation and the immune system, contributing to the early pathogenesis of cutaneous SCC (Nindl, Gottschling, and Stockfleth 2007).

**FIGURE 1:**
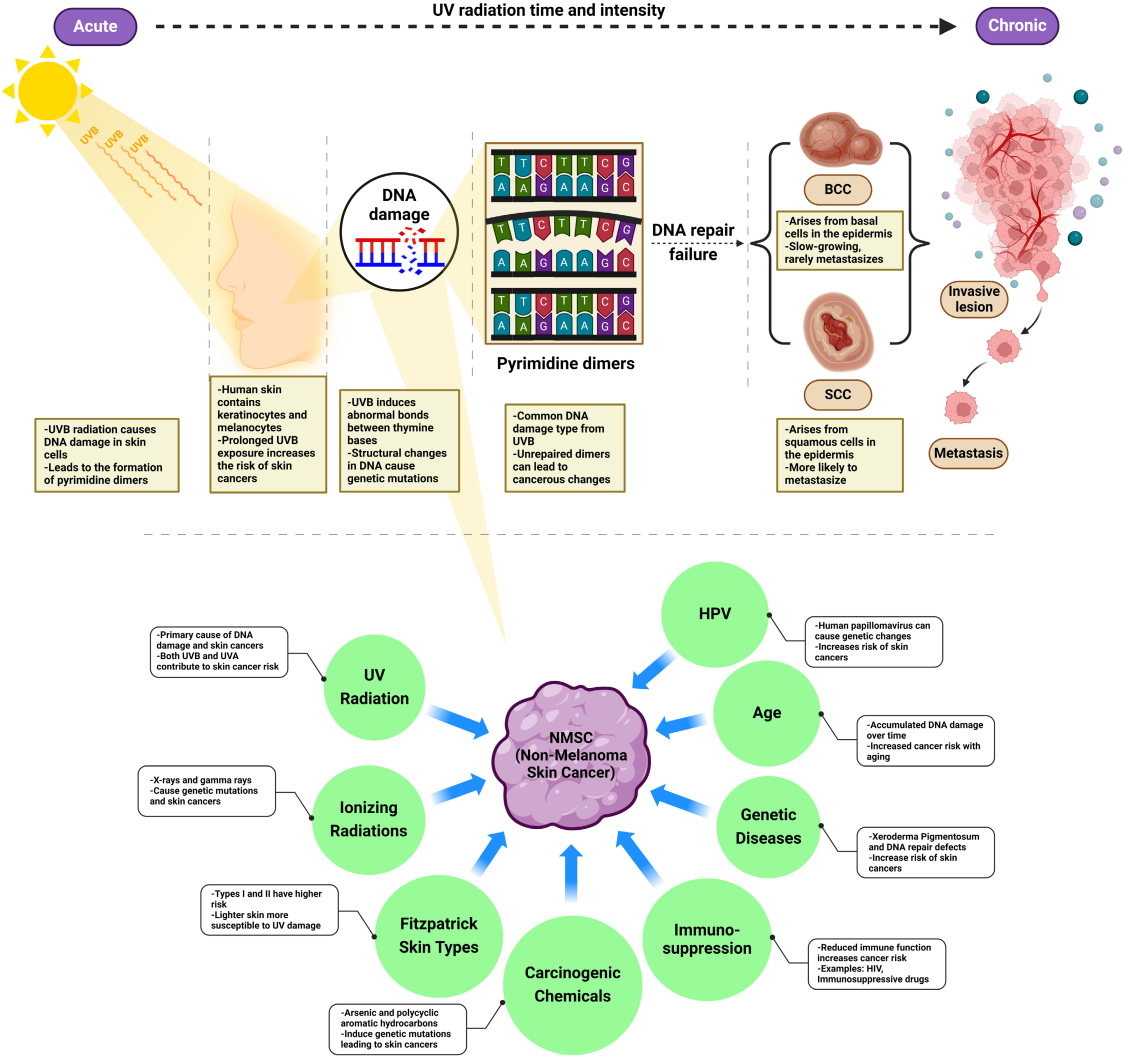
Factors involved in NMSC pathogenesis. UV radiation is a primary cause, leading to DNA damage and the formation of pyrimidine dimers, which can result in genetic mutations if not properly repaired. Immunosuppression, whether due to medical conditions or immunosuppressive drugs, increases the risk of NMSC by reducing the body's ability to repair damaged DNA. Additionally, exposure to carcinogenic chemicals, including arsenic and polycyclic aromatic hydrocarbons, can contribute to the pathogenesis of NMSC. Age is another factor, as accumulated DNA damage over time increases the likelihood of cancer development. HPV infections can cause genetic changes that elevate the risk of skin cancers. Lastly, Fitzpatrick skin types I and II, which are more susceptible to UV damage, have a higher risk of developing NMSC.

NMSC is primarily driven by genetic and molecular alterations induced by UV radiation, leading to somatic mutations characteristic of UV signatures (Didona et al. 2018; Yoshioka et al. 2022). Key genes implicated in the pathogenesis of NMSC include the vitamin D receptor (VDR) and retinoid X receptor (RXR), with differential expression patterns observed in various stages of skin lesions (Ocanha‐Xavier et al. 2022). Genetic polymorphisms in the TGF‐β and VEGF‐A genes also modulate susceptibility to NMSC, with certain alleles offering protective effects while others increase risk (Scola et al. 2022). Whole‐exome sequencing has identified pathogenic mutations in genes such as Kras, Fat1, and Kmt2c, which are potential drivers of SCC in UV‐exposed, DNA repair‐deficient models (Yoshioka et al. 2022). The interplay between genetic factors and environmental exposure underscores the complexity of NMSC pathogenesis and the need for targeted prevention and therapeutic strategies (Zambrano‐Román et al. 2022). Figure 1 shows the genetic factors and other factors affecting the development of NMSC (Didona et al. 2018; Zambrano‐Román et al. 2022).

Several treatment options exist for managing SC, including surgical procedures, radiation therapy, topical chemotherapy, immunotherapy, and targeted therapy (Anand et al. 2022; Zeng et al. 2023). Topical chemotherapy is widely utilized among these treatment approaches due to its cost‐effectiveness and convenience (Capanema et al. 2018). 5‐Fluorouracil (5‐FU) is a frequently utilized drug in FDA‐approved topical chemotherapy and has been a preferred choice among clinicians for an extended period due to its potent anticancer effects against SC (Cunningham et al. 2017; Hao et al. 2020). Additionally, the administration of postsurgical chemotherapy medications frequently results in various adverse effects, coupled with substantial treatment expenses. Consequently, investigating novel and efficacious SC therapies utilizing natural medicine holds critical clinical and societal importance (Chinembiri et al. 2014; Sajadimajd et al. 2020).

Natural compounds such as curcumin, apigenin, quercetin, and resveratrol (RES) have shown promising effects in the prevention and treatment of NMSC. These compounds exhibit anti‐inflammatory, antitumor, and antioxidant properties that help mitigate the harmful effects of UV radiation, which is a major risk factor for NMSC (Peterle et al. 2023; Afaq and Katiyar 2011).

RES, identified chemically as 3, 5, 4′ trihydroxystilbene, is a naturally derived biphenolic substance highly concentrated in grape skins and seeds and present in various plant‐based edibles and drinks like peanuts, berries, and red wine (Kaur et al. 2021; Kumar et al. 2021).

RES has shown promise in treating atopic dermatitis (AD) and psoriasis due to its anti‐inflammatory properties. It can inhibit mast cell activation, suppress inflammatory signaling pathways, and reduce the production of proinflammatory cytokines (Marko and Pawliczak 2023; Carlucci et al. 2023). RES exerts antiaging effects by enhancing skin elasticity, density, and smoothness. It can help reduce skin roughness and redness, contributing to a more youthful appearance (Janssens‐Böcker and Kerscher 2021).

Resveratrol's chemopreventive properties are attributed to its ability to modulate various molecular pathways involved in the initiation and progression of cancer. It influences pathways such as NF‐κB, MAPK, and PI3K‐AKT, which are critical in cell proliferation, apoptosis, and inflammation (Borriello 2017). By modulating these pathways, RES can prevent the early stages of carcinogenesis, such as DNA damage and mutations.

Additionally, RES has demonstrated antioxidant capabilities, aiding in counteracting the impacts of reactive oxygen species (ROS) induced by UV radiation, thereby reducing DNA damage and mutation (Tong et al. 2015; Brand et al. 2018). Furthermore, RES has been reported to couple apoptosis with autophagy, providing a multifaceted approach to combating SC (Szulc‐Musioł and Sarecka‐Hujar 2021). Studies indicate that RES might augment the effectiveness of chemotherapy drugs, such as 5‐FU, in the treatment and control of skin carcinoma (Iqubal, Iqubal, Anjum, et al. 2021; Iqubal, Iqubal, Imtiyaz, et al. 2021). In vitro research demonstrates that RES can effectively hinder the growth and spread of diverse tumor cell types, including human cutaneous SCC A431 cells, and induce apoptosis (Zhang et al. 2018; Zhai et al. 2016). Additionally, in vivo studies using nude mice with xenograft models of human skin SCC A431 demonstrated that RES significantly inhibited tumor growth (Hao et al. 2013). This inhibitory effect was associated, leading to an escalation in the expression of p53 and ERK and a reduction in the expression of survivin, ultimately leading to apoptosis of tumor cells (Hao et al. 2013). RES was found to significantly reduce the UV‐B‐induced elevated regulation of Ki‐67 protein levels, which is a marker of proliferation associated with cancer development (Aziz, Afaq, and Ahmad 2005). Kim et al. (2011) showed that oral RES administration in mice led to a delay in UV‐triggered skin tumor development and a reduction in the malignant conversion of benign papillomas to SCCs. Additionally, their research highlighted the increased expression of TGF‐β2 in UV‐induced SCCs and its subsequent suppression following RES treatment.

Back et al. (2012) identified an increase in Rictor, an element of the mTORC2 signaling assembly, in murine SCCs induced by UV light. This elevation is mitigated through oral RES administration.

In summary, RES has demonstrated promising potential in preventing and treating NMSC through its ability to inhibit UV‐B‐mediated phototoxicity, modulate cellular pathways involved in cancer development, and enhance the efficacy of other cancer treatments when combined with a lipid nanosystem for dermal delivery.

Additionally, resveratrol's diverse pharmacological effects, combined with advancements in drug delivery technologies, make it a promising candidate for drug development. Further research and clinical trials are necessary to fully establish its safety, efficacy, and optimal dosing for various medical conditions.

This study aims to elucidate the effects of RES on NMSC, focusing on its mechanisms of action, efficacy in preclinical studies, and potential as a chemopreventive and therapeutic agent. The review specifically examines RES's influence on oxidative stress, apoptosis, and cell proliferation in NMSC. It also discusses the enhancement of RES's bioavailability and therapeutic effects through advanced delivery systems like lipid nanosystems, nanoparticles, and other nanocarriers. The review underscores the need for comprehensive clinical analyses to fully understand RES's effectiveness and safety in humans and to optimize delivery methods for improved therapeutic outcomes.

## 2 Overview of Resveratrol

### 2.1 Structure

**FIGURE 2:**
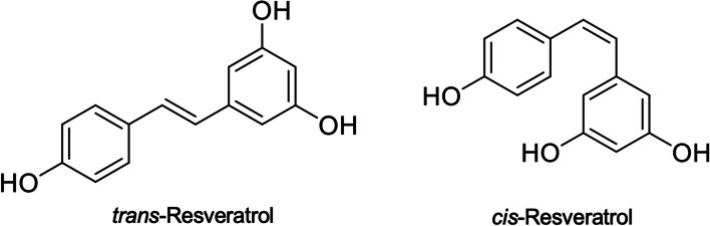
Chemical structure of RES (Meng et al. 2023).

RES is a polyphenolic constituent characterized by its two phenolic rings linked through a styrene double bond (Pecyna et al. 2020). It is also known as 3, 4′, 5 trihydroxystilbene and is a hydroxylated form of stilbene, belonging to the stilbene class of polyphenols (Perrone et al. 2017). The molecular structure of RES allows for the formation of two isomers: Trans and cis. The trans variant is more stable and predominant (Figure 2) (Camont et al. 2009).

### 2.2 Sources

RES was first isolated by Michio Takaoka in 1939 from Veratrum grandiflorum O. Loes, commonly known as white hellebore. It is abundantly found in the dried roots of *Polygonum cuspidatum* and in the skins and seeds of grape berries, especially in red wine (Hu et al. 2013; Li et al. 2006). Besides grapes, RES is also present in berries, including blueberries and cranberries, which contribute to its dietary intake (Olson et al. 2021). Grape berries contain RES in varying amounts, ranging from 0.16 to 3.54 μg/g (Frémont 2000). Studies have shown that red wine has a significantly higher concentration of polyphenolic compounds compared to white wine (Stervbo, Vang, and Bonnesen 2007). In red wine, the concentration of RES ranges from 0.1 to 15 mg/L, while in white wine, it spans from 0.1 to 2.1 mg/L (Frémont 2000; Mukherjee, Dudley, and Das 2010).

### 2.3 Solubility and Improving Bioavailability

RES is known for its health benefits but is significantly limited by its poor water solubility and low bioavailability (Febres‐Molina, Prat‐Resina, and Jaña 2023; Liu et al. 2023). The solubility of RES is a critical factor affecting its therapeutic efficacy and potential applications in medicine (Harwansh, Yadav, and Deshmukh 2023). The aqueous solubility of RES is below 0.05 mg/mL, which affects its uptake and biological availability (Springer and Moco 2019).

Various strategies have been developed to enhance the solubility of RES, including the use of nanotechnology and chemical modifications (Harwansh, Yadav, and Deshmukh 2023; Li et al. 2023).

One approach involves the use of glucosylation, where RES is modified by enzymes like glucosyltransferase to improve its solubility. This enzymatic modification leads to the formation of water‐soluble RES derivatives, which can enhance its bioavailability (Febres‐Molina, Prat‐Resina, and Jaña 2023).

Another method to improve RES solubility is through the development of cocrystals with different coformers, such as piperazine, which have shown increased aqueous solubility (Mehta et al. 2018). Nanocarriers, including liposomes and nanoparticles, have also been explored as delivery systems to improve the solubility and bioavailability of RES (Harwansh, Yadav, and Deshmukh 2023). For instance, folate‐modified liposomes have been used to enhance the uptake and therapeutic effects of RES in cancer treatment (Zhu et al. 2023). Gold nanoparticles functionalized with peptides have been utilized to control the release of RES, thereby improving its solubility and targeted delivery in cancer therapy (Liu et al. 2023). Additionally, RES can be loaded onto mesoporous silica nanoparticles, which act as carriers to enhance its solubility and bioavailability (Ioniţă et al. 2022).

These nanoparticles provide a controlled release mechanism, allowing RES to be delivered effectively to target sites (Ioniţă et al. 2022; Summerlin et al. 2016). The use of carbon dots derived from RES has also been investigated to improve its solubility and enhance its wound‐healing properties (Cheng et al. 2023). These carbon dots exhibit excellent biocompatibility and have shown promising results in accelerating wound healing in vivo (Cheng et al. 2023). Furthermore, fibrinogen, a protein involved in blood coagulation, has been found to increase the solubility of RES by binding to it, thus preventing oxidation and enhancing its antioxidative properties (Gligorijević et al. 2020). This interaction between RES and fibrinogen highlights the potential for using protein interactions to improve solubility and bioavailability (Gligorijević et al. 2020).

Despite these advancements, the solubility of RES remains a significant challenge that continues to drive research in the development of novel delivery systems and chemical modifications (Harwansh, Yadav, and Deshmukh 2023). The exploration of different nanocarriers and chemical derivatives is crucial for overcoming the limitations of resveratrol's solubility and enhancing its therapeutic potential. As research progresses, the development of more efficient and effective methods to improve RES solubility will likely lead to better clinical outcomes and broader applications in medicine (Harwansh, Yadav, and Deshmukh 2023).

Phytoglycogen has been used to enhance the solubility and in vitro permeation of RES, thereby improving its bioavailability (Chen and Yao 2023). Water‐in‐Oil‐in‐Water (W/O/W) emulsions improve the physicochemical stability and digestion resistivity of RES, facilitating better transport and absorption in the gastrointestinal tract (Shi, Wang, Guo, et al. 2021).

To enhance RES's antitumor potential, blending it with other medications or treatments like curcumin (Narayanan et al. 2009), quercetin (Caddeo et al. 2016), paclitaxel (Meng et al. 2016), docetaxel (Singh, Lillard Jr, and Singh 2018), and 5‐fluorouracil (Cosco et al. 2015) proves effective.

The study by Narayanan et al. (2009) investigated the effects of liposome‐encapsulated curcumin and RES on prostate cancer incidence in PTEN knockout mice. The researchers aimed to enhance the bioavailability of these phytochemicals, which are known for their poor absorption, by using liposome encapsulation. The combination of liposomal curcumin and RES significantly reduced the incidence of prostatic adenocarcinoma in vivo, as evidenced by a marked decrease in tumor presence in the treated mice. In vitro assays demonstrated that the combination effectively inhibited cell growth and induced apoptosis in PTEN‐CaP8 cancer cells.

The study by Caddeo et al. (2016) explored the effects of co‐incorporating quercetin and RES in liposomes to combat the inflammatory and oxidative responses associated with skin cancer. The researchers found that this combination significantly reduced oxidative stress and inflammation, which are key contributors to skin cancer progression. The liposomal formulation enhanced the bioavailability and stability of quercetin and RES, leading to more effective antioxidant and anti‐inflammatory activities. In vitro experiments demonstrated that the liposomal formulation could protect skin cells from oxidative damage and reduce the production of proinflammatory cytokines. These findings suggest that liposome‐encapsulated quercetin and RES could be a promising therapeutic strategy for managing skin cancer by mitigating inflammation and oxidative stress.

In another study, Meng et al. (2016) examined the effectiveness of using liposomes to co‐encapsulate RES and paclitaxel for reversing drug resistance in breast cancer cells. The combination therapy demonstrated a significant improvement in overcoming multidrug resistance in breast cancer cells compared to using paclitaxel alone. In vivo experiments showed that the co‐encapsulation strategy enhanced the therapeutic efficacy of paclitaxel, leading to a substantial reduction in tumor growth. They also found that the liposomal formulation improved the bioavailability and stability of both RES and paclitaxel, contributing to the enhanced antitumor effects. These results suggest that co‐encapsulating RES and paclitaxel in liposomes could be a promising approach to improve treatment outcomes in drug‐resistant breast cancer.

The study by Singh, Lillard Jr, and Singh (2018) focused on reversing drug resistance in prostate cancer using nanoparticles loaded with RES and docetaxel, prepared through planetary ball milling (PBM). The researchers observed that the PBM nanoparticles significantly enhanced the cytotoxic effects of docetaxel on drug‐resistant prostate cancer cells compared to docetaxel alone. In vivo experiments demonstrated that the nanoparticle formulation led to a substantial reduction in tumor size, indicating improved therapeutic efficacy. They also found that the combination of RES and docetaxel in PBM nanoparticles effectively modulated drug resistance mechanisms, such as the downregulation of efflux transporters. These results suggest that PBM nanoparticles co‐loaded with RES and docetaxel could be a promising strategy for overcoming drug resistance in prostate cancer therapy.

The study by Cosco et al. (2015) investigated the use of ultradeformable liposomes as carriers for the topical delivery of RES and 5‐fluorouracil. The researchers found that these liposomes significantly improved the skin penetration of both drugs, which are known for their limited permeability when applied topically. The co‐encapsulation of RES and 5‐fluorouracil in ultradeformable liposomes enhanced their stability and allowed for a controlled release, leading to prolonged therapeutic effects. In vitro experiments demonstrated that this formulation effectively inhibited the growth of cancerous cells, suggesting a potential for treating skin cancer. The study concluded that ultradeformable liposomes could be a promising strategy for the topical delivery of multiple drugs, improving their bioavailability and therapeutic efficacy.

Exploring the combined effect of RES with commonly used cancer drugs, such as docetaxel and doxorubicin, on solid tumor cell lines (MCF‐7, HeLa, and HepG2) in vitro revealed the increased cytotoxicity of these drugs while reducing side effects like cardiotoxicity (Al‐Abd et al. 2011). It was noted that the simultaneous use of RES with docetaxel and doxorubicin enhanced the expression levels of Bax and Bcl‐2 (Al‐Abd et al. 2011). RES also enhanced the tumor‐suppressing effect of fluorouracil on murine hepatoma22 cells by causing a halt in the S phase of the tumor cells (Wu et al. 2004). Additionally, RES significantly reduced the toxicity associated with fluorouracil (Wu et al. 2004).

Encasing RES within mesoporous silica nanoparticles significantly improved its solubility and controlled release in vitro, resulting in approximately 95% improvement (Juère et al. 2017). Furthermore, integrating RES with gold and silver nanoparticles amplified its antibacterial and anticarcinogenic properties (Park et al. 2016). Recently, when RES was encapsulated in lecithin, known as nano‐RES, it proved effective in combating breast cancer in vitro, specifically targeting BT474 human breast cancer cells (Liang et al. 2022).

Cutting‐edge vectors, such as polymer‐derived nanoparticles, liposomal carriers, micellar structures, and metallic nanoparticles, enhance RES's robustness, dissolution, and ability to traverse cell membranes. This optimization allows RES to more precisely attack cancerous cells (Yang et al. 2020; Sharifi‐Rad et al. 2021). The synergistic combination of RES with other chemotherapeutic agents has shown efficacy in increasing their lethal impact on cancer cells while mitigating negative reactions. Innovations like incorporating RES in nanoporous colloidal silica or within lecithin matrices highlight its viability as an effective oncological agent, particularly in nanoscale formulations. However, a recent scholarly review elaborates on the need for comprehensive clinical analyses of nano‐RES against a spectrum of malignancies, including multiple myeloma, colorectal, hepatic, and neuroendocrine neoplasms (Sarfraz et al. 2023).

### 2.4 Limitations of Nanotechnology in Resveratrol Delivery

Complex Manufacturing Processes: The production of nanoparticles involves complex techniques that can be costly and challenging to scale up for commercial use (Paliwal, Babu, and Palakurthi 2014).

Potential Toxicity: The long‐term safety and potential toxicity of nanoparticles remain concerns. Rigorous testing and regulatory standards are necessary to ensure their safe use in humans (Ettlinger et al. 2022; Yildirimer et al. 2011).

Stability Issues: Nanoparticles can face stability issues in biological environments, which may affect their efficacy and safety (Sharma et al. 2014).

### 2.5 Absorption

Studies using human intestinal epithelial cell lines, such as Caco‐2 cells, have demonstrated efficient absorption of resveratrol in vitro (Kaldas, Walle, and Walle 2003; Maier‐Salamon et al. 2006). Transport across the Caco‐2 monolayer occurs in a direction‐independent manner, with permeability values being much higher than those of paracellular transport markers, suggesting efficient absorption in vivo (Kaldas, Walle, and Walle 2003).

Studies have consistently shown that about 75% of an oral dose of resveratrol is absorbed in humans. The primary mechanism of absorption is believed to be passive diffusion across the intestinal epithelium (Walle et al. 2004; Mattio et al. 2019; Brockmueller et al. 2024). After an oral dose of 25 mg in healthy human subjects, the plasma concentrations showed significantly higher bioavailability of RES metabolites (approximately 2 μM) compared to native RES (40 nM), indicating a notable difference (Walle et al. 2004; Goldberg, Yan, and Soleas 2003). Studies have shown that administration of higher oral doses of RES, from 2 to 5 g, results in peak plasma concentrations greater than 10 μM (la Porte et al. 2010; Howells et al. 2011; Boocock et al. 2007).

After absorption, RES enters the bloodstream, where it binds to plasma proteins such as albumin for transport. It can also associate with lipoproteins, including very low‐density lipoproteins (VLDL) and high‐density lipoproteins (HDL), facilitating its distribution throughout the body (Rocha et al. 2011).

### 2.6 Metabolism

The intestine and liver are described as major sites of metabolism for RES (Springer and Moco 2019).

In the intestine, RES undergoes conjugation reactions, primarily glucuronidation and sulfation, within enterocytes (Planas et al. 2012). In the liver, RES undergoes phase II metabolism, primarily through conjugation reactions (Springer and Moco 2019):

1. Glucuronidation by UDP‐glucuronosyltransferases (UGTs), especially UGT1A1 and UGT1A9
2. Sulfation by sulfotransferases (SULTs), especially SULT1A1

The major metabolites formed in the liver are RES glucuronide and sulfate conjugates, which are formed by UDP‐glucuronosyltransferases and sulfotransferases, respectively. RES‐3‐O‐glucuronide and RES‐3‐O‐sulfate are the predominant metabolites found in human plasma and urine (Planas et al. 2012; Wenzel and Somoza 2005).

The gut microbiota also plays a significant role in RES metabolism. The microbiota can transform RES through processes such as double‐bond reduction, dihydroxylation, and demethylation, resulting in metabolites that may have different or even stronger biological activities than the parent compound (Jarosova et al. 2019). This metabolic transformation highlights the importance of the gut microbiota in modulating the bioavailability and efficacy of RES. The endoplasmic reticulum (ER) plays a crucial role in both VLDL assembly and the metabolism of various compounds in the liver (Gilham et al. 2005). This shared location increases the possibility of interaction between RES and the VLDL assembly process. Further research specifically focusing on resveratrol's interaction with VLDL assembly in the hepatic ER would be needed to confirm this hypothesis. The current evidence primarily demonstrates resveratrol's effects on the overall liver lipid metabolism rather than its direct incorporation into VLDL particles.

Micronized RES refers to a form of RES that has been processed to reduce its particle size to less than 5 μm (SRT501). This reduction in particle size is believed to enhance the bioavailability of RES primarily due to the increase in surface area and improved suspension properties, which can lead to better absorption across the gastrointestinal tract and thus higher systemic availability of the parent compound (Howells et al. 2011).

In colorectal cancer patients, micronized RES at a daily dose of 5 g for a duration of 10 to 21 days demonstrated significantly enhanced bioavailability compared to nonmicronized RES. The peak serum concentration of micronized RES tripled, reaching 8.51 μM, compared to 2.40 μM for nonmicronized RES (Howells et al. 2011; Boocock et al. 2007).

### 2.7 Transport Into Cells

Resveratrol's transport into cells is a complex process influenced by various molecular mechanisms. One key mechanism involves its interaction with cellular transport proteins, such as organic anion‐transporting polypeptides (OATPs), which facilitate the uptake of RES and its metabolites into cells (Riha et al. 2014). These transporters, including OATP1B1, OATP1B3, and OATP2B1, have been shown to significantly enhance the cellular uptake of RES, suggesting their crucial role in its bioavailability (Riha et al. 2014). Additionally, resveratrol's lipophilic nature allows it to interact with cell membranes, potentially facilitating passive diffusion into cells. However, this passive transport is often limited by its rapid metabolism and poor solubility. To overcome these challenges, RES can be encapsulated in nanocarriers, such as liposomes and nanoparticles, which enhance its solubility and protect it from degradation, thereby improving its cellular uptake (Maleki Dana et al. 2022; Chung et al. 2020). RES can permeate cells through passive diffusion and form complexes with integrins (Delmas et al. 2011). In addition to its ability to permeate cells, RES can interact with integrins, which are transmembrane receptors that facilitate cell–extracellular matrix adhesion. Specifically, RES has been shown to interact with integrin αvβ3 in breast cancer cells. This interaction is significant because integrin αvβ3 serves as a receptor for nonpeptide hormones, and resveratrol's binding to this integrin can mediate its antiproliferative and pro‐apoptotic effects in cancer cells (Ho et al. 2020).

### 2.8 Skin Uptake of RES

The skin uptake of RES through topical administration is a critical aspect of its therapeutic application, particularly in dermatological treatments and skin health. To enhance the topical bioavailability of RES, various strategies have been employed, including the incorporation of RES into nanocarriers such as solid lipid nanoparticles (SLN), NLC, and ultradeformable liposomes (UDL). These delivery systems have shown promise in improving the skin's uptake of RES, thereby maximizing its therapeutic potential in various dermatological applications (Intagliata et al. 2019; Chen et al. 2017). Chen et al. (2017) developed RES‐loaded NLC utilizing cetyl palmitate as the solid lipid and sesame oil as the liquid lipid to enhance resveratrol's skin permeation. Following in vitro percutaneous absorption tests on human skin, they observed an increased skin permeation of RES from these NLC, indicating that these nanocarriers could be effectively used to improve the topical effectiveness of RES (Chen et al. 2017).

One study assessed the effectiveness of RES‐loaded SLN in gel formulations for treating irritant contact dermatitis (ICD) (Shrotriya, Ranpise, and Vidhate 2017). The researchers conducted both in vitro skin permeation tests on human skin and in vivo experiments on BALB/c mice with induced ICD to evaluate the formulation's ability to enhance RES skin penetration and reduce ear swelling. The findings showed that SLN increased RES retention in the skin layers (epidermis and dermis) by approximately 3 times compared to free RES. Additionally, the gel containing RES‐loaded SLN was as effective as a commercial corticosteroid‐based formulation in reducing tissue edema. Based on these results, the authors concluded that RES‐loaded SLN could be a viable therapeutic option for treating ICD (Shrotriya, Ranpise, and Vidhate 2017).

The topical administration of RES can be significantly improved through the use of advanced drug delivery systems and chemical modifications.

### 2.9 Toxicity

Even at high concentrations, such as 1000–1500 mg/day, mammals show good tolerance to RES (Sergides et al. 2016). Research confirms that consuming 100–1000 mg/day of RES is safe as a dietary polyphenol, supported by in vivo animal models and clinical studies (Vesely, Baldovska, and Kolesarova 2021; Turner et al. 2015; Wang and Sang 2018). RES is considered safe and nontoxic even at high doses of up to 5 g (Cottart, Nivet‐Antoine, and Beaudeux 2014). Clinical trials have shown that consuming 2.5–5 g of RES daily can cause mild to moderate gastrointestinal issues (Brown et al. 2010), with diarrhea observed when consumed twice daily at 2 g (la Porte et al. 2010). It is worth noting that micronized versions of RES have been associated with improved tolerability (Smoliga and Blanchard 2014). Studies have shown that RES can reduce chemotherapy‐related nonhematological toxicities, indicating its role in improving the tolerability of cancer treatments (Ostwal et al. 2022; Ren et al. 2021). RES influences cell proliferation and has been shown to have varying effects depending on the cell type. In cancer biology, it exhibits cytotoxic effects on malignant cells while sparing nonmalignant cells (Jovanović Galović et al. 2022; Fulda 2010). The effects of RES on the stem cell population, particularly human mesenchymal stem cells (hMSCs), at chronic doses are complex and depend on the concentration and duration of exposure (Peltz et al. 2012; Yin et al. 2020). At higher concentrations, RES has been shown to induce cellular senescence and irreversible cell cycle arrest in proliferating MSCs, potentially limiting their therapeutic potential (Shaito et al. 2020; Hu and Li 2019). Long‐term intake of RES can act as a thyroid disruptor and a goitrogen, which may negatively impact stem cell function and overall health (Shaito et al. 2020). Despite its potential benefits, the pro‐oxidant effects associated with high‐dose RES administration may lead to oxidative stress in stem cells, potentially compromising their viability and regenerative capacity.

The claim regarding the nontoxicity of chronic high‐dose RES intake, such as 5 g/day, is a topic of ongoing research and debate (Patel et al. 2011). One of the concerns with high‐dose RES intake is its potential to induce oxidative stress through the activation of Phase II detoxification enzymes, which could lead to liver damage. These enzymes, while generally involved in detoxification processes, can sometimes produce reactive intermediates that contribute to oxidative stress and cellular damage. Recent studies have explored the protective effects of RES against oxidative stress and liver damage, often focusing on its ability to modulate the Nrf2‐Keap1 signaling pathway, which plays a crucial role in cellular antioxidant defense mechanisms (Ding et al. 2023; Shahcheraghi et al. 2023).

The safety of RES in humans requires further investigation through well‐designed clinical trials to establish comprehensive safety profiles and understand any long‐term effects. Additionally, the potential for RES to interact with other drugs or compounds necessitates careful consideration, especially in combination therapies. The development of novel formulations and delivery systems aims to enhance the therapeutic index of RES while minimizing any potential risks. As research progresses, understanding the molecular mechanisms underlying resveratrol's effects will be crucial in optimizing its use and ensuring safety in clinical applications. Overall, while RES is generally safe, further studies are needed to confirm its safety and efficacy in diverse populations and clinical settings.

## 3 Chemopreventive and Anticancer Activities of Resveratrol Against NMSC

### 3.1 Mechanism of Resveratrol‐Induced Apoptosis

Apoptosis plays a crucial role in SC (Strozyk and Kulms 2013). In normal physiological processes, apoptosis helps eliminate damaged or mutated cells, preventing cancer development (Kiraz et al. 2016). However, in SC, the balance of apoptosis is disrupted, resulting in reduced cell death and increased survival of cancerous cells (Labi and Erlacher 2015). This imbalance contributes to uncontrolled growth and proliferation of cancer cells, ultimately promoting SC progression (Wang et al. 2018). Therefore, aberrations in programmed cell death significantly influence the emergence and development of cutaneous malignancy (Plati, Bucur, and Khosravi‐Far 2008). Recent studies have shown that prolonged elevation of ROS levels in cancer cells have tumor‐inhibitory properties and trigger apoptotic sequences (Feehan and Shantz 2016; Sahoo et al. 2022). The occurrence of NMSC can be influenced by factors such as anatomic localization, UV exposure, and senescence. Apoptosis and immune response also play a role in this process (Manestar‐Blažić et al. 2007). Understanding the signaling cascades that initiate apoptosis will provide insights into the pathogenesis of NMSC.

5‐FU, a sanctioned medication, is known for its powerful ability to treat cutaneous malignancy (de Oliveira et al. 2021). It works by blocking DNA synthesis in cancer cells through inhibiting thymidylate synthase, which leads to accelerated apoptosis and reduced cell proliferation (Smith et al. 2020). 5‐FU is commonly used in cancer chemotherapy and is effective in stopping the growth of cancer cells (Sethy and Kundu 2021). Its effectiveness in managing various cancers, including SC, has been well‐documented (Vodenkova et al. 2020).

Caspase‐3 and Ki‐67 are important markers in the context of SC (Iqubal, Iqubal, Anjum, et al. 2021). Caspase‐3 is a key protein involved in the process of apoptosis (Asadi et al. 2022). In SC, the level of cleaved caspase‐3 indicates the extent of apoptosis. An increase in its level indicates a pro‐apoptotic effect, leading to cancerous cell death (Chang et al. 2021). On the other hand, Ki‐67 is a marker for tumor proliferation (Menon et al. 2019). An elevated level directly reflects the degree of cellular proliferation, while a diminished level indicates the drug's antiproliferative impact (Mansy et al. 2020). Therefore, assessing cleaved caspase‐3 and Ki‐67 levels provides valuable insights into the apoptotic and antiproliferative abilities of therapeutic interventions (Bhutia et al. 2016).

Survivin, an essential protein, plays a crucial role in regulating cellular survival and apoptosis (Chen et al. 2016). It belongs to the inhibitor of apoptosis (IAP) gene family and is involved in inhibiting apoptosis, promoting cellular proliferation, and managing the cell cycle (Shamsabadi et al. 2016). Survivin is notably overexpressed in various cancers and is associated with increased cancer aggression and reduced patient survival rates (Santarelli et al. 2018). Studies have shown its involvement in the development and progression of cutaneous malignancies, including BCC, SCC, and melanoma (Khan et al. 2017). Survivin is also implicated in hindering papilloma regression and promoting its transition to SCC (Frassanito et al. 2019). Therefore, it has become a prime target for investigation in cancer treatment strategies (Mobahat, Narendran, and Riabowol 2014).

Smac/DIABLO is a crucial mitochondrial component that coordinates apoptotic processes (Liu et al. 2013). It counteracts the inhibitory effect of IAPs on apoptosis (Creagh et al. 2004). In response to pro‐apoptotic signals, Smac/DIABLO moves from the mitochondria to the cytoplasm. Once there, it interacts with IAPs, facilitating caspase activation and driving the apoptotic pathway (Paul et al. 2018).

Aziz et al. (2005) conducted a study that demonstrated RES's significant chemopreventive properties against UVB‐induced SC in SKH‐1 hairless mice. They found that RES's preventive effects are in part due to changes in survivin's expression and function. Furthermore, their results indicate that RES enhances apoptosis in UV‐B‐induced skin tumors, suggesting its potential for eliminating damaged or precancerous cells. The study also proposed a mechanism by which resveratrol's chemopreventive effects occur, involving the suppression of Thr34 phosphorylation in survivin, which leads to a decrease in survivin levels and an increase in Smac/DIABLO expression. Ultimately, this leads to the programmed elimination of abnormal or cancerous cells. These findings open up possibilities for developing RES‐based solutions to combat dermatological malignancies and conditions caused by UV radiation.

In their investigation, Aziz, Afaq, and Ahmad (2005) assessed the efficacy of RES in mitigating the impacts of UV‐B radiation on dermal health in rodent models. The study revealed that RES has the ability to suppress UV‐B‐induced cellular hyperplasia, reduce the expression of proteins implicated in oncogenesis (specifically epidermal cyclooxygenase‐2 and ornithine decarboxylase), and decrease survivin levels, a protein that inhibits apoptosis. This research highlighted the antiproliferative properties of RES, particularly against the proliferative response triggered by UV‐B exposure. Frequent exposure to UV‐B significantly increased the expression of Ki‐67 protein, indicating enhanced cellular proliferation. However, RES preconditioning effectively counteracted this increase in Ki‐67 expression caused by UV‐B, suggesting that it has the potential to inhibit UV‐B‐induced cellular proliferation. Furthermore, RES reversed the decrease in Smac/DIABLO protein and enhanced UV‐B‐induced apoptosis. The investigation also showed that RES protects against UV‐B‐induced dermal damage by inhibiting survivin and related processes. This research suggests that RES could be beneficial in preventing UV‐B‐related skin impairments, including skin tumorigenesis. It calls for more comprehensive studies to evaluate the effectiveness of RES in preventing skin tumorigenesis caused by UV exposure.

Liu et al. (2017) demonstrated that RES at a concentration of 100 μM effectively inhibited the proliferation of Colo16 SCC cells and induced apoptosis. This effect was characterized by a decrease in Ki‐67 levels, the presence of apoptotic cell features, and an increased proportion of apoptotic cells following RES exposure. Moreover, the inhibition of cell growth and induction of apoptosis by RES can be attributed to the suppression of the canonical Wnt signaling pathway and a reduction in the expression of key Wnt target genes that are crucial for human SCCs. Additionally, their study revealed an upregulation of Axin2 expression, an antagonist of the Wnt signaling pathway, after RES treatment. Furthermore, they discovered that transfection with a specific siRNA targeting β‐catenin increased the susceptibility of Colo16 cells to RES, suggesting that the effects of resveratrol on SCC cells are not solely dependent on Wnt signaling and may involve other important cancer‐related factors. Overall, these findings support the idea that RES inhibits the proliferation of squamous cell carcinoma cells and suppresses Wnt signaling, providing valuable insights for potential therapeutic strategies in treating epidermal SCCs.

5‐Aminolevulinic acid‐induced photodynamic treatment is a noninvasive method for treating various tumors, including SC (Shi, Wang, Chen, et al. 2021). ALA is a photosensitizer that accumulates in hyperproliferating tumor cells. When activated by a specific wavelength of light, it generates ROS, triggering irreversible destruction of the tumor (Capriglione 2021). This therapy is highly effective in treating premalignant and malignant SCs, as well as other tumors like lung and colon cancer (Casas 2020). ALA‐PDT offers several advantages, including accurate targeting of tumor tissues, noninvasiveness, favorable cosmetic outcomes, fewer adverse effects, short therapy duration, and reduced expenses compared to alternative treatments (Komolibus et al. 2021). However, its efficacy may be reduced for expansive or deeply situated tumors, and resistant or recurrent tumors may emerge (Mastrangelopoulou et al. 2022).

The MAPK/ERK pathway, known as the mitogen‐activated protein kinase/extracellular signal‐regulated kinase cascade, plays a pivotal role in various cellular mechanisms, including cell growth, differentiation, and survival (Moon and Ro 2021). This pathway is crucial in cancer development and progression (Asl et al. 2021). Activation of the MAPK/ERK pathway can lead to malignant cell transformation, uncontrolled proliferation, and neoplastic expansion (Asl et al. 2021).

p38, also known as p38 MAPK, serves as a key signal transducer in cellular responses to different stressors such as UV radiation, thermal stress, and inflammatory cytokines (Yang et al. 2014). As a member of the MAPK family, it regulates cell growth, differentiation, and apoptosis (Sun et al. 2015). Activating the p38 pathway can induce programmed cell death and suppress cell growth, making it a significant focus in oncological therapies (Martínez‐Limón et al. 2020).

Zhang et al. (2018) discovered that RES has a significant effect on the growth of A431 cells, suppressing it and inducing apoptosis. When combined with ALA‐PDT, RES further enhances the inhibition of cell proliferation and increases the rate of apoptosis in these cells. Moreover, treatment with RES activates the MAPK pathway, resulting in heightened phosphorylation of both MAPK/ERK and MAPK/p38 in A431 cells. This activation of the MAPK pathway is believed to be crucial in facilitating apoptosis in A431 cells. The researchers also found that the p38 inhibitor SB203580 reduces the impact of RES on A431 cells, suggesting the involvement of the MAPK/p38 pathway in the enhanced anticancer effects of RES and ALA‐PDT. Therefore, this study suggests that RES can be used as an adjunct to enhance the anticancer efficacy of ALA‐PDT by inhibiting cell proliferation and promoting apoptosis through the stimulation of the MAPK pathway.

The S‐phase, or synthesis phase, is a stage in the cell cycle during which DNA replication occurs (Darzynkiewicz et al. 2015). This phase is crucial in cellular mitosis as it involves duplicating genetic content in preparation for cell division. During the S‐phase, the cell's DNA is replicated to ensure that each resulting daughter cell inherits an identical genetic blueprint (Ovejero, Bueno, and Sacristán 2020). The S‐phase plays a fundamental role in cellular growth and proliferation (Celis and Celis 1985).

The study by Dun et al. (2015) demonstrated that the concurrent use of RES and 5‐FU has a synergistic effect in suppressing cancer cell proliferation and promoting apoptosis. This synergy was observed in both laboratory and animal studies. In the animal model, the dual therapy significantly reduced the total tumor count. At the cellular level, RES induced an S‐phase arrest, increased the proportion of cells undergoing apoptosis, and enhanced the expression of apoptotic proteins such as caspase‐3, cleaved PARP, p53, Bax, and Bcl‐2. These findings suggest that combining RES with 5‐FU holds promise as a potentially less harmful strategy for cancer treatment. The research highlights the potential benefits of integrating natural dietary compounds with conventional chemotherapy agents to improve cancer treatment outcomes.

These findings indicate that RES may have potential chemopreventive effects on NMSC through its influence on apoptosis. However, the specific molecular signaling cascades involved in RES‐induced apoptosis in NMSC are not fully understood. Further research is needed to fully comprehend the effects of RES on apoptosis in the context of NMSC.

miR‐126 is a microRNA that plays an important role in controlling gene expression (Li et al. 2018). Research has shown that miR‐126 is associated with the regulation of angiogenesis, vascular integrity, and cancer progression (Nammian et al. 2020). In fact, it is considered a potential tumor suppressor in different types of cancer, and its dysregulation has been linked to the development and progression of cancer (Jalil et al. 2023).

The Wnt/β‐catenin signaling cascade is a crucial cellular mechanism that plays a role in various biological functions, including embryonic growth, tissue equilibrium, and cellular division (Lorzadeh et al. 2021). This pathway is essential for controlling gene transcription and cellular activity (Rennoll and Yochum 2015). When the Wnt signaling pathway is activated, β‐catenin becomes stabilized and accumulates in the cytoplasm (Lorzadeh et al. 2021). Then, β‐catenin moves to the nucleus, where it works together with transcription factors to regulate the expression of specific genes involved in cellular proliferation, motility, and diversification (Bian et al. 2020).

Zhang and Najafi (2020) investigated the effects of RES on skin SCC and its underlying mechanisms. Their findings demonstrated that RES effectively inhibited the proliferation, movement, and invasiveness of HSC‐5 cells. These effects were correlated with an increase in miR‐126 expression and a decrease in β‐catenin protein levels. Upregulation of miR‐126 was found to reduce the proliferation, migration, and invasion of CSCC cells, while downregulation had the opposite effect. The study also showed that reducing miR‐126 expression counteracted the suppressive effects of RES on CSCC cell behavior and β‐catenin protein levels. These results suggest that the inhibitory actions of RES on CSCC cells may be linked to increased miR‐126 levels, leading to the inhibition of the Wnt/β‐catenin signaling pathway. Overall, this study highlights the potential therapeutic benefits of RES in the prevention and treatment of CSCC, emphasizing the importance of miR‐126 and the Wnt/β‐catenin signaling pathway in mediating RES's effects on CSCC cells.

Hao et al. (2013) reported that RES had a significant suppressive effect on the proliferation of human skin SCC A431 xenografts in nude mice. Their study showed that RES treatment resulted in a concentration‐dependent decrease in tumor growth, with higher doses producing a stronger suppression. In addition, RES was found to increase the levels of p53 and ERK, while decreasing the expression of survivin, leading to apoptosis in the tumor cells. Histological evaluations revealed pronounced tumor necrosis and a clear distinction between necrotic and tumor tissues after RES treatment. Moreover, RES treatment significantly increased the apoptotic index (AI) compared to the control groups. These results suggest that RES has the potential to be a therapeutic option for inhibiting the growth of human skin SCC.

### 3.2 Anti‐Inflammatory Action and Mechanisms of Resveratrol

Inflammatory cytokines play a critical role in the onset and advancement of cutaneous malignancy (Neagu et al. 2019). Continuous exposure to UV radiation is known to increase the production of ROS and pro‐inflammatory cytokines, leading to inflammation and promoting the initiation and progression of cutaneous malignancy (Ciążyńska et al. 2021). High levels of pro‐inflammatory cytokines such as TNF‐α, IL‐6, and IL‐1α, along with low levels of anti‐inflammatory cytokines like IL‐10, are associated with the inflammatory response in SC (Neagu et al. 2019). These cytokines can regulate oncogene levels, contribute to angiogenesis, and play a crucial role in tumor invasion and metastasis (Aguilar‐Cazares et al. 2019). The imbalance between pro‐inflammatory and anti‐inflammatory cytokines is a contributing factor in the development and progression of cutaneous malignancy (Markovics et al. 2021).

Iqubal, Iqubal, Anjum, et al. (2021) established the effectiveness of a lipid‐nanosystem‐based gel called linogel, which contains 5‐FU and RES, in treating cutaneous malignancy. In their study, they evaluated the therapeutic potential of linogel in vivo by measuring the tumor count, area, and volume. The findings revealed that linogel demonstrated strong antioxidant and anti‐inflammatory properties. This was evident through the regulation of oxidative stress indicators such as SOD, CAT, and GSH, as well as inflammatory cytokine concentrations including TNF‐α, IL‐6, and IL‐10. The research also highlighted the heightened antioxidant activity of RES in linogel. This activity effectively neutralized ROS in the affected region, prevented DNA alteration, and expedited DNA repair. These findings suggest that the combination of RES and 5‐FU in the form of linogel effectively mitigated oxidative stress and inflammation associated with SC. Furthermore, the study evaluated the apoptosis‐inducing and antiproliferative effects of linogel by estimating the levels of cleaved caspase‐3 and ki‐67. The results indicated that linogel exhibited potent apoptotic and antiproliferative effects compared to conventional gel formulations. Specifically, linogel significantly elevated the level of cleaved caspase‐3, indicating increased apoptotic activity. Additionally, there was a reduction in the level of ki‐67, which signifies an antiproliferative effect. These findings suggest that the developed linogel effectively induced cancerous cell death and inhibited cell proliferation, making it a promising therapeutic approach for the treatment of SC. The study also highlighted the enhanced apoptotic and antiproliferative effects of the combination of 5‐FU and RES in the form of linogel, further supporting its potential as an effective treatment for SC.

### 3.3 Effects of Resveratrol on Oxidative Stress

The potential of RES as a chemopreventive agent for NMSC is implied by its ability to reduce oxidative stress. Higher levels of ROS, including hydrogen peroxide (H_2_O_2_), hydroxyl radical (OH^−^), and oxygen radicals (O^−^), are associated with the reduced activity of antioxidant enzymes like SOD, CAT, and GSH (Das and Roychoudhury 2014). This imbalance leads to the excessive accumulation of harmful radicals, leading to oxidative stress (Pizzino et al. 2017). Adequate levels of these antioxidant enzymes are crucial for protecting DNA from damage, reducing ROS buildup, preventing genetic mutations, and suppressing the activation of genes that promote cell death, thus serving as a protective factor against cancer formation (Vostrikova, Grinev, and Gogvadze 2020). Furthermore, numerous studies have demonstrated the carcinogenic potential of ROS, while antioxidant supplementation has been shown to have tumor‐inhibiting effects (Momtaz, Hassani, and Abdolghaffari 2022). Therefore, oxidative stress plays a crucial role in the occurrence and progression of SC, driven by mechanisms such as DNA disruption, genetic mutations, and the upregulation of oncogenes (Iqubal and Haque 2022).

In NMSC, prolonged exposure to UV radiation can weaken the antioxidant defense, which may contribute to the development of multiple stages of cancer (Sander et al. 2003). Overall, chronic exposure to environmental factors that generate ROS and cause oxidative stress in the skin plays a significant role in the pathogenesis of NMSC.

RES has been shown to have protective properties against oxidative damage in NMSC. It acts as a potent antioxidant and can inhibit oxidative DNA damage caused by UV radiation (Ndiaye et al. 2011).

Caddeo et al. (2016) conducted a study in which liposomes were used to simultaneously deliver quercetin and RES, resulting in a significant reduction in skin swelling and myeloperoxidase activity in TPA‐induced (TPA: An inducer of inflammation and oxidative stress) skin lesions in mice. The liposomes facilitated the penetration of quercetin and RES into fibroblasts, leading to strong anti‐ROS and anti‐inflammatory effects. These effects were particularly important in the context of squamous cell carcinoma (SC), as they demonstrated a dose‐dependent decrease in cellular ROS levels and a significant reduction in swelling and infiltration of leukocytes, which are associated with premalignant and malignant skin conditions. Moreover, the liposomal formulations reduced the potential harmful effects of quercetin and RES, especially when used together, and improved the overall stability of these compounds. Furthermore, the liposomal delivery method enhanced the therapeutic efficacy of quercetin and RES, particularly in attenuating oxidative stress and inflammation associated with premalignant and malignant skin disorders. These findings suggest that the combined liposomal administration of quercetin and RES could be beneficial in the treatment of skin conditions resulting from inflammation and oxidative damage.

In summary, the potential of resveratrol as a chemopreventive agent for NMSC is implied by its ability to reduce oxidative stress and promote apoptosis.

### 3.4 Other Mechanisms of Resveratrol

The A431 cell line is a well‐established human epithelial carcinoma cell line commonly used in cancer research and drug development (Hernández‐Alcoceba, Fernández, and Lacal 1999). These cells were originally derived from a vulvar SCC and are known for their high expression of epidermal growth factor receptors (EGFR) (Kim et al. 2009). A431 cells are commonly used in research focused on cancer biology, drug screening, and assessing anticancer agents due to their significance in understanding the molecular underpinnings of cancer and evaluating potential therapeutic approaches (Memariani et al. 2021).

In Iqubal, Iqubal, Imtiyaz, et al.'s (2021) research, the impact of RES on the A431 cell line was analyzed, particularly when combined with 5‐FU, to explore its potential anticancer activity. The findings showed that the lipid‐nanosystem, specifically the blend of RES and 5‐FU, exhibited significantly enhanced effectiveness on the A431 cell line compared to a standard formulation. This suggests that the combination of RES and 5‐FU, delivered through the lipid‐nanosystem, may have enhanced cytotoxic effects on the A431 cell line, potentially indicating a synergistic or additive effect in inhibiting the growth of cancerous cells.

Nanostructured lipid carrier (NLC) gel, a lipid‐oriented drug delivery system, is engineered to optimize the delivery of therapeutic agents to the skin (Elmowafy and Al‐Sanea 2021). NLCs are composed of a lipid binary mixture and a surfactant, and they are characterized by their smaller particle size, disordered crystal structure, and high drug payload (Sharma and Baldi 2018). These characteristics offer advantages such as enhanced stability, reduced drug leakage, and ease of formulation compared to other lipid formulations like SLN and liposomes (Mohammed et al. 2023). The small size of NLCs facilitates intimate skin interaction, promoting increased drug penetration (Czajkowska‐Kośnik, Szekalska, and Winnicka 2019). When formulated into a gel, NLCs form a single lipid layer on the skin, inhibiting transepidermal water loss and enhancing skin moisture content (Eroğlu, Sinani, and Ülker 2023).

In Imran et al.'s (2020) research, NLCs were used to administer a blend of quercetin and RES for the treatment of SC. The aim was to improve the distribution of the blend in the dermal and epidermal layers of the skin. The NLCs had a particle size of 191 nm ± 5.20. The study compared the cytotoxic effects of the NLC‐based gel with a standard gel on A431 cells using the MTT assay. Different concentrations of both gels were applied for 24 h, and cytotoxicity was assessed thereafter. The results showed that the NLC gel had higher cytotoxicity, with a lower IC50 value compared to the standard gel. Specifically, the IC50 of the NLC gel was 86.50 μM, while the IC50 of the standard gel was 123.64 μM. This highlights the enhanced effectiveness of the NLC gel in combating SC cells. The investigation also confirmed that the NLC gel effectively delivered quercetin and RES into deeper layers of the skin, indicating its potential as a therapeutic agent for SC.

Ursolic acid (UA), an organic compound found in various sources such as apples and rosemary (Waheed et al. 2021), has been shown to inhibit tumor formation and decrease the survival of tumor cells in different contexts, including cutaneous malignancies (Prasad et al. 2016). However, some malignancies do not respond to UA interventions (Lin et al. 2020).

P‐glycoprotein (P‐gp), an ATP‐dependent transporter, plays a crucial role in conferring resistance to chemotherapy drugs in both experimental and clinical settings (Yang and Liu 2016). This protein, encoded by the ABCB1 gene, consists of two halves, each with six transmembrane segments and an ATP‐binding domain. The transcriptional activity of P‐gp is regulated by various elements related to multidrug resistance (MDR), including NFκB (Tian et al. 2023). P‐gp is overexpressed in many cancer types, allowing the removal of various pharmaceuticals and thereby promoting chemotherapeutic resistance (Tian et al. 2023). Its high presence in healthy and malignant cells of the liver, kidney, and colon is a major factor in the inherent resistance of these tumors to chemotherapy (Heming et al. 2022). Additionally, P‐glycoprotein concentrations increase in hematological and breast malignancies as the tumor progresses or in response to chemotherapy treatments, resulting in reduced treatment effectiveness (Gote et al. 2021). Studies on P‐glycoprotein antagonists, conducted through in vitro and in vivo experiments as well as some clinical trials, have demonstrated their potential to enhance the efficacy of standard chemotherapy drugs (Cao et al. 2020). The investigation of P‐gp inhibitors is a promising approach to overcome multidrug resistance in cancer therapies (Zhang et al. 2021).

RES enhances the antidermal oncogenic properties of UA through a collaborative interaction, which is different from the suppression of P‐glycoprotein (P‐gp)‐facilitated UA efflux. Research has shown that skin carcinoma cells (Ca3/7) have increased tolerance to UA‐induced cell death and higher levels of P‐gp. When used together with UA, RES improves the effectiveness of UA in both cell lines. Furthermore, RES has been observed to enhance cancer cells' response to conventional chemotherapy agents by promoting apoptotic mechanisms and inhibiting P‐gp‐driven efflux. The combined effect of RES and UA operates through a different pathway compared to P‐gp inhibitors, suggesting that RES strengthens UA's antiskin carcinoma properties through an alternative mechanism (Junco et al. 2013). This suggests that combining UA with RES could be an effective strategy against skin carcinoma.

TGF‐β2 is a key protein involved in the progression of epithelial tumors (Singh et al. 2023). It belongs to the TGF‐beta family, which includes TGF‐β1, TGF‐β2, and TGF‐β3 (Massagué and Sheppard 2023). TGF‐beta2 is known for its increased expression in both human malignancies and various animal cancer models, including SCCs (Latil et al. 2017). Its higher presence is associated with the more advanced stages of cancer development, and it contributes to the enhancement of epithelial–mesenchymal transition (EMT), tumor progression, and spread (Bévant et al. 2022). The regulation of TGF‐β2 is controlled by various signaling networks, particularly the Smad‐dependent and Smad‐independent pathways (Massagué and Sheppard 2023), and its expression is influenced by agents such as protein kinase B (Akt) and CREB (Oshimori and Fuchs 2012).

In their study, Kim et al. (2011) investigated the effects of RES as an antineoplastic agent, targeting the TGF‐β2 signaling axis to inhibit UV‐triggered tumor progression. Using both in vivo and in vitro techniques, their study revealed the impact of RES on the development of skin neoplasms and the invasive behavior of SCC cells. The findings showed that oral administration of RES in mice delayed the onset of UV‐induced skin neoplasia and reduced the transition of benign papillomas into SCCs. RES notably inhibited both the TGF‐β2/Smad‐dependent and independent pathways, leading to reduced invasiveness of A431 cells. Furthermore, RES suppressed the activation of Akt and CREB, which are important regulators of TGF‐β2 expression. This evidence supports the idea that RES can effectively suppress tumor progression and invasiveness by modulating the TGF‐β2 pathway, thus providing potential avenues for the management and treatment of invasive carcinomas.

Macroautophagy, commonly known as autophagy, is a cellular mechanism that plays a critical role in degrading and recycling intracellular components (Nieto‐Torres and Hansen 2021). This process begins with the formation of an autophagosome, which is a specialized structure that encloses cytoplasmic materials targeted for decomposition (Galluzzi et al. 2017). The autophagosome then merges with a lysosome, creating an autolysosome where lysosomal enzymes break down the enclosed cellular matter (Lőrincz and Juhász 2020). Autophagy is essential for maintaining cellular balance by removing unnecessary, defective, or aging proteins and organelles (Papackova and Cahova 2014). Interestingly, autophagy has a dual role in cancer, as it can either support the survival of cancer cells or act as a tumor suppressor, depending on the specific cellular context (Chavez‐Dominguez et al. 2020).

Rictor, a key component of the mTORC2 signaling complex, plays a vital role in various cellular processes, including cell proliferation, survival, and cytoskeleton organization (Zou et al. 2016; Ramaiah and Kumar 2021).

In their groundbreaking research, Back et al. (2012) investigated the impact of RES on autophagy and its significance in countering the development of squamous cell carcinoma (SC) induced by UV radiation. They found that RES induces premature senescence in human skin cells, inhibiting the formation of autolysosomes. This interruption is linked to a significant decrease in the expression of Rictor, a component of mTORC2, which subsequently leads to changes in the actin cytoskeleton. Remarkably, their investigations also showed that Rictor is overexpressed in UV‐induced SCCs in mice and that its expression is significantly reduced after oral administration of RES. These findings demonstrate that RES interferes with the autophagic pathway by modulating Rictor, and this downregulation of Rictor emerges as a potential mechanism for tumor suppression, closely associated with the induction of premature senescence. This study not only provides insights into the underlying mechanisms of RES's actions but also opens up new possibilities for utilizing autophagy modulation in the development of innovative cancer therapies.

Human telomerase reverse transcriptase (hTERT) is a crucial enzyme involved in preserving telomeres, which are essential structures found at the ends of chromosomes (Plyasova and Zhdanov 2021).

Acting as a unique chromosomal terminal transferase, telomerase's primary function is to add repetitive nucleotide sequences to chromosome ends, counteracting the loss of telomeric DNA during cell division (Giardini et al. 2014). This activity is critical for maintaining the chromosome length and stability, thereby preventing the depletion of genetic information during cellular replication (Zheng et al. 2014). The hTERT protein, which serves as the catalytic component of telomerase, is closely associated with telomerase activation (Yuan, Larsson, and Xu 2019). In many malignant tumors, including hTERT, there is an increase in telomerase activity, contributing to uncontrolled cell proliferation (Guterres and Villanueva 2020).

The study examined the effects of RES on human A431 epidermoid carcinoma cells. The findings showed that RES significantly inhibited the proliferation of A431 cells in a dose‐dependent manner. Additionally, RES reduced the telomerase activity and decreased the expression of the hTERT protein in a concentration‐dependent manner. RES also induced morphological changes in A431 cells, indicative of apoptosis. This research provides a theoretical basis for considering RES as a potential clinical intervention for squamous cell carcinoma. The evidence suggests that RES can effectively downregulate hTERT protein expression and hinder telomerase activity in A431 cells, essential processes in curbing their proliferation (Zhai et al. 2016).

RES is being investigated for its potential role in cancer therapy due to its ability to suppress tumor invasion and metastasis by inhibiting signaling pathways associated with cancer progression. It has been shown to inhibit the proliferation of various human tumor cells in laboratory studies and has been linked to reductions in inflammation, angiogenesis, and metastasis (Bishayee 2009). These findings suggest that RES may hold promise in the prevention and treatment of NMSC, although further research, including clinical trials, is necessary to fully understand its effectiveness and safety in humans. RES is generally considered safe when taken in small amounts for a short period. However, at doses of 2.5 g or more per day, side effects such as nausea, vomiting, diarrhea, and liver dysfunction may occur, especially in patients with nonalcoholic fatty liver disease (Shaito et al. 2020; Salehi et al. 2018).

**FIGURE 3:**
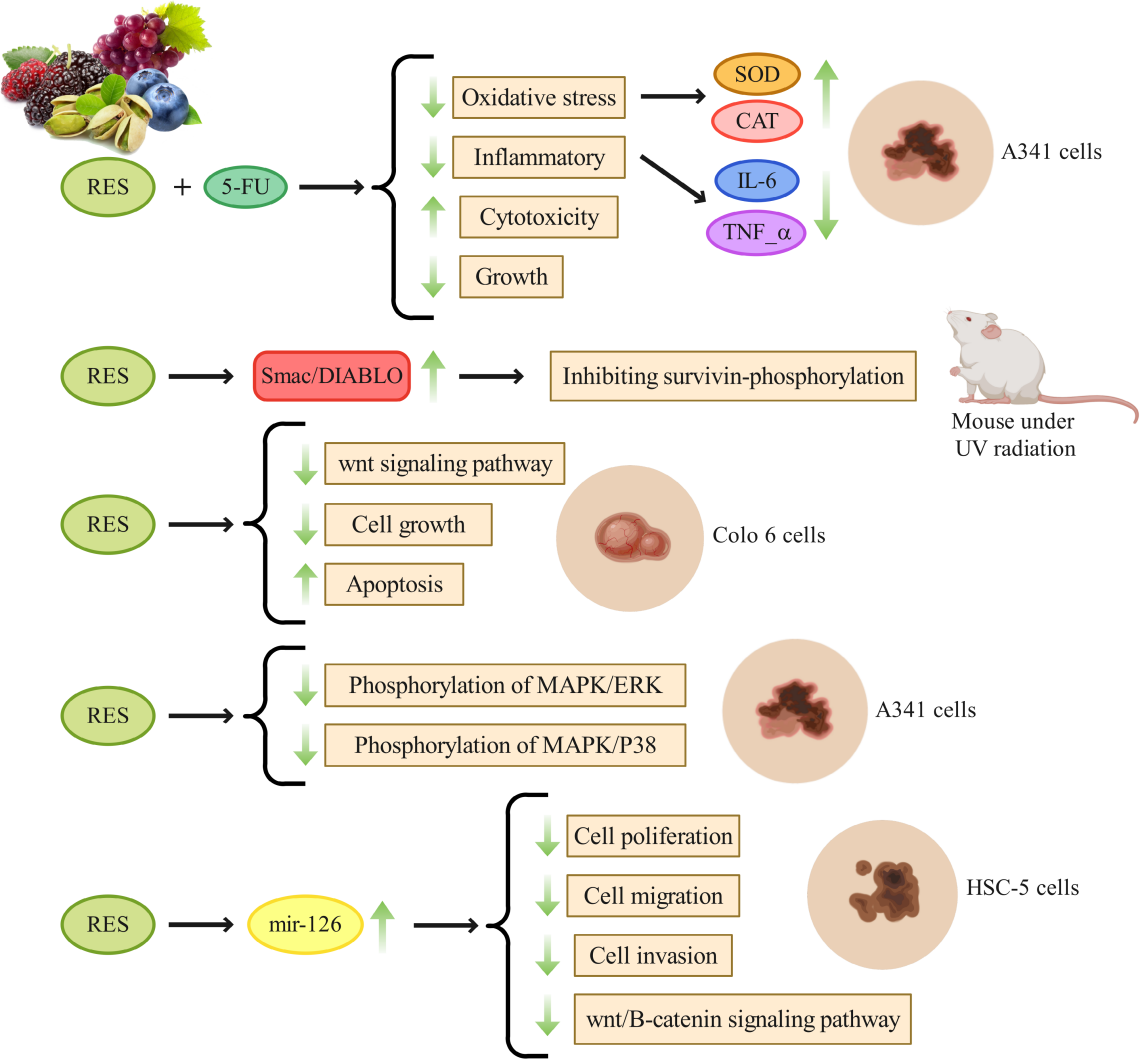
Anticancer role of RES against NMSC: Focusing on oxidative stress, cellular, and molecular mechanisms. RES exhibits chemopreventive and antineoplastic capabilities against NMSC. It does so by triggering apoptosis in cancerous cells, inhibiting proliferation, and regulating inflammatory and oxidative stress pathways. The research highlighted in the manuscript indicates that a synergistic lipid‐nanocarrier incorporating RES and 5‐FU showed significantly enhanced effectiveness in curtailing the proliferation of malignant cells, notably in the A431 cell line, compared to traditional formulations. This figure indicates that RES increases the expression of mir‐126, which in turn leads to decreased cell proliferation, migration, and invasion, as well as inhibition of the Wnt/β‐catenin signaling pathway.

Table 1 and Figure 3 present a summary of the results from previous studies on the anticancer activities of RES against NMSC.

**TABLE 1:** Anticancer activities of resveratrol against NMSC.

Authors	Dosage of resveratrol (RES)	Type of model	Mechanisms
Iqubal et al.	0.5 mg of each drug per gram gel, topically	Mice	The combination therapy (RES+ 5‐FU) demonstrated an antitumor effect by increasing S‐phase arrest and tumor apoptosis, thus suggesting the potential of this approach to improve the skin cancer treatment option
Imran et al.	10 mg	A431 cells	The NLC gel showed promising results in cytotoxicity assays, with a lower IC50 compared to the conventional gel, demonstrating its potential as an effective vehicle for delivering RES for the treatment of skin cancer. Therefore, the study evidenced that RES, formulated in the NLC gel, could serve as a promising strategy for the treatment of skin cancer
Aziz et al.	50 μM (topically in 200 μL acetone) topically	Mice	RES imparted strong chemopreventive effects against UVB exposure‐mediated skin carcinogenesis, possibly mediated through the modulation of survivin and associated event
Aziz et al.	10 μmol in 200 μL acetone, topically	Mice	Pretreatment with RES resulted in significant inhibition of UV‐B exposure‐mediated increases in cellular proliferation, protein levels of epidermal cyclooxygenase‐2 and ornithine decarboxylase, and protein and messenger RNA levels of survivin in the skin of SKH‐1 hairless mice
Liu et al.	100 μM	Colo16 cells	RES inhibited the cell growth and induced apoptosis in Colo16 cells, potentially through the inactivation of Wnt signaling. This inhibition of Wnt signaling may have contributed to the observed effects on cell growth and apoptosis, highlighting the potential therapeutic impact of RES on human epidermal SCCs
Dun et al.	50 μmol topically	TE‐1 and A431cells	RES increased the accumulation of S‐phase cells susceptible to 5‐FU, potentially enhancing the sensitivity of tumor cells to 5‐FU
Caddeo et al.	25, 50 and 100 μM, topically	Mice	The study concludes that the liposomal co‐delivery of quercetin and RES holds promise for the treatment of skin cancer by mitigating the associated inflammatory and oxidative responses (scavenging of ROS)
Zhang et al.	40 μmol/L	HSC‐5 cells	RES intervention, the growth rate, migration, and invasion of HSC‐5 cells were significantly reduced, accompanied by an increase in miR‐126 expression and a decrease in β‐catenin protein expression. Upregulating miR‐126 also resulted in a significant reduction in the growth rate, migration, and invasion of HSC‐5 cells
Hao et al.	20 and 40 μg/g	Mice	RES has an inhibitory effect on xenografts and indicates that its inhibitory mechanism involves an increase in p53 expression and a decrease in SVV expression, consequently inducing the apoptosis of tumor cells
Kim et al.	200 mg/kg, oral gavage	Mice	RES treatment resulted in p53‐independent suppression of UVB‐induced skin carcinogenesis in p53+//SKH‐1 mice

## 4 Conclusions and Future Perspectives

RES has shown promising chemopreventive and anticancer abilities against NMSC through multiple pathways. Its protective role against oxidative stress in NMSC is noteworthy, as it possesses strong antioxidant properties that can counteract UV‐induced oxidative DNA damage. Moreover, RES induces apoptosis in NMSC cells, inhibits their proliferation, and affects inflammatory and oxidative stress pathways. Research also suggests that RES may enhance the effectiveness of chemotherapeutic agents like 5‐FU in SC therapy. Both laboratory and animal studies have observed RES's ability to suppress tumor cell growth and progression, delay UV‐induced SC development, and reduce the likelihood of benign papillomas progressing into SCCs. These findings highlight RES's potential as a chemopreventive agent for NMSC. Additionally, RES seems to synergize with compounds such as UA to enhance anti‐SC effects. Advanced delivery systems, including polymeric nanoparticles, liposomes, and metallic nanoparticles, are being investigated to improve the stability and efficacy of RES in cancer treatment. Overall, these findings position RES as a promising therapeutic candidate for the prevention and treatment of NMSC.

## Author Contributions

**Mohammad Yasin Zamanian:** conceptualization (equal), investigation (equal), project administration (equal), writing – review and editing (equal). **Taha Shahbazi:** writing – original draft (equal). **Syeda Wajida Kazmi:** writing – original draft (equal). **Beneen M. Hussien:** resources (equal). **Abhishek Sharma:** methodology (equal). **Maytham T. Qasim:** writing – original draft (equal). **Ahmed Hjazi:** data curation (equal), investigation (equal). **Ibrohim B. Sapaev:** resources (equal). **Ayda Nouri Danesh:** writing – original draft (equal). **Niloofar Taheri:** writing – original draft (equal), writing – review and editing (equal). **Maryam Golmohammadi:** conceptualization (equal), investigation (equal), project administration (equal), supervision (equal), visualization (equal), writing – original draft (equal), writing – review and editing (equal).

## Ethics Statement

The authors have nothing to report.

## Consent

The authors have nothing to report.

## Conflicts of Interest

The authors declare no conflicts of interest.

## Data Availability

The authors have nothing to report.
